# Microencapsulation of Propolis by Complex Coacervation with Chia Mucilage and Gelatin: Antioxidant Stability and Functional Potential

**DOI:** 10.3390/antiox14070845

**Published:** 2025-07-10

**Authors:** Carlos A. Ligarda-Samanez, David Choque-Quispe, Henry Palomino-Rincón, Elibet Moscoso-Moscoso, Rodrigo J. Guzmán Gutiérrez, Ismael Banda Mozo

**Affiliations:** 1 Nutraceuticals and Biomaterials Research Group, Universidad Nacional José María Arguedas, Andahuaylas 03701, Peru; dchoque@unajma.edu.pe (D.C.-Q.); hpalomino@unajma.edu.pe (H.P.-R.); emoscoso@unajma.edu.pe (E.M.-M.); 1008820181@unajma.edu.pe (R.J.G.G.); 1003320212@unajma.edu.pe (I.B.M.); 2 Agroindustrial Engineering School, Universidad Nacional José María Arguedas, Andahuaylas 03701, Peru; 3 Food Nanotechnology Research Laboratory, Universidad Nacional José María Arguedas, Andahuaylas 03701, Peru; 4 Research Group in the Development of Advanced Materials for Water and Food Treatment, Universidad Nacional José María Arguedas, Andahuaylas 03701, Peru; 5 Water and Food Treatment Materials Research Laboratory, Universidad Nacional José María Arguedas, Andahuaylas 03701, Peru

**Keywords:** spray drying, controlled release, phenolic compounds, functional gummies, thermal stability

## Abstract

Propolis is a bee-derived resin rich in phenolic compounds known for their antioxidant, anti-inflammatory, and antimicrobial properties; however, its limited solubility and stability hinder its incorporation into food matrices. This study aimed to optimize the microencapsulation of ethanolic propolis extract through complex coacervation using chia mucilage and gelatin as wall materials, followed by spray drying. A 3^2^ factorial design was applied to evaluate the effects of coacervate concentration and inlet temperature on various microcapsule properties. The optimal formulation (3.13% coacervate and 120 °C) exhibited high phenolic retention (15.36 mg GAE/g), notable antioxidant capacity (60.10 µmol TE/g), good solubility, thermal stability, and sustained in vitro release. Phenolic compounds were identified and quantified by UPLC-PDA-QDa, including gallic acid, catechin, epicatechin, epigallocatechin gallate, rutin, myricetin, resveratrol, quercetin, and kaempferol. Incorporating the microcapsules into functional gummy candies significantly enhanced their antioxidant activity without compromising sensory attributes. These findings support the use of complex coacervation as an effective strategy for stabilizing propolis bioactives, with promising applications in the development of functional foods that offer potential health benefits.

## 1. Introduction

Beekeeping has undergone significant advancements in recent years, enabling the production of a range of products, including honey, pollen, royal jelly, and propolis. The latter, in particular, is of great interest due to its bioactive compounds, which have recognized health benefits [1,2,3]. Propolis is a resinous substance collected by honeybees (*Apis mellifera*) from the surrounding flora. It is noted for its remarkable antioxidant, anti-inflammatory, and antimicrobial properties, which are highly valued in the food, pharmaceutical, and cosmetic industries [4,5]. However, the direct application of propolis faces substantial limitations due to its poor solubility and low bioavailability, which restrict its effectiveness in practical applications [2,4,6].

Microencapsulation has been utilized as an effective method to preserve the bioactive properties of propolis, thereby expanding its applicability in various industries. Techniques such as spray drying, emulsification, and freeze-drying have been employed to protect its phenolic compounds; however, they present limitations, including low encapsulation efficiency, uncontrolled release, and poor colloidal stability [5,7,8]. Given these challenges, complex coacervation has garnered growing attention as a promising alternative, as it enables the formation of microcapsules through electrostatic interactions between wall biopolymers, thereby enhancing antioxidant retention and facilitating sustained release under controlled conditions [9,10]. However, a knowledge gap remains regarding the use of combined natural biopolymers, such as chia mucilage and gelatin, which represent an innovative strategy for stabilizing propolis bioactives in functional products.

Chia mucilage (*Salvia hispanica* L.) is an anionic polysaccharide with a complex structure, gelling and emulsifying properties, and film-forming abilities due to its high water retention capacity and the presence of uronic functional groups. These features make it a promising plant-based wall material for controlled release systems. Gelatin, on the other hand, is a positively charged animal-derived protein that forms stable complexes with polysaccharides in acidic media, making it widely used in complex coacervation processes. It provides structural cohesion, good solubility, and compatibility with various industrial applications. The synergistic combination of both biopolymers allows the formation of spherical microcapsules with high encapsulation efficiency (above 90%) and suitable colloidal stability, supporting their use as a functional encapsulation system in diverse food matrices [11,12,13].

In this context, this study aimed to evaluate the microencapsulation of phenolic extracts from propolis through complex coacervation and spray drying, using a mixture of chia mucilage and gelatin as wall materials. The hypothesis was that this combination would improve the physicochemical stability, controlled release, and antioxidant functionality of propolis. To achieve this, a factorial design was applied to optimize process conditions, and the resulting microcapsules were characterized through physicochemical, instrumental, and functional analyses. Additionally, their application in a functional gummy-type food matrix was validated by evaluating both sensory acceptance and functional performance. Ultimately, this work contributes to the advancement of clean and sustainable technologies for the valorization of beekeeping products.

## 2. Materials and Methods

### 2.1. Materials

Propolis samples were collected in the Chumbao River Valley, in the locality of Cuncataca (Andahuaylas, Apurímac, Peru), and were kindly provided by the Chumbao River Beekeepers Association. Chia seeds and red prickly pears were purchased from the municipal market of Andahuaylas, and the gelatin used was of commercial origin (passionfoods, Lima, Peru; manufactured in China). The following reagents and auxiliary materials were used in the study, organized according to their function:

Reagents for phenolic compound and antioxidant analysis: Folin–Ciocalteu reagent (Himedia, Mumbai, India), gallic acid (Sigma-Aldrich, Taufkirchen, Germany), quercetin (Sigma-Aldrich, Taufkirchen, Germany), aluminum chloride hexahydrate (Sigma-Aldrich, Taufkirchen, Germany), Trolox [6-hydroxy-2,5,7,8-tetramethylchroman-2-carboxylic acid] (Sigma-Aldrich, Taufkirchen, Germany), DPPH [2,2-diphenyl-1-picrylhydrazyl] (Himedia, Mumbai, India), and ABTS [2,2′-azino-bis(3-ethylbenzothiazoline-6-sulfonic acid)] (Sigma-Aldrich, Taufkirchen, Germany).

Salts and buffers: sodium carbonate (Na_2_CO_3_), potassium persulfate (K_2_S_2_O_8_), and sodium chloride (NaCl), all from commercial sources (Spectrum, New Brunswick, NJ, Canada).

Solvents: 96% ethanol (Coderal, Lima, Peru) and 99% isopropyl alcohol (Martell, Lima, Peru).

Analytical standards for chromatography: gallic acid, catechin, epicatechin, epigallocatechin gallate, rutin, myricetin, resveratrol, quercetin, and kaempferol (Sigma-Aldrich, Taufkirchen, Germany).

Ingredients for the formulation of functional gummy candies: glucose (Jofsac, Lima, Peru), isomalt (passionfoods, Lima, Peru; manufactured in Germany), potassium benzoate, glycerin (Alkofarma, Lima, Peru), and commercial sucrose.

### 2.2. Extraction of Chia Mucilage

The chia seeds were first sorted to remove any visible debris and impurities. They were then soaked in distilled water at a ratio of 1:25 (*w*/*v*), stirring constantly for 4 to 5 h at 40 °C in a water bath (WTB50, Memmert, Germany, Schwabach). During this time, the seeds absorb water, causing the outer mucilaginous layer to expand. Once optimal hydration was achieved, the mucilage was separated from the liquid by filtration using a fine cloth. The extract obtained was dried in a forced convection oven (FED 115, Binder, Germany, Tuttlingen) at 50 °C for 24 h. It was then ground using a cyclone impact mill (Retsch Cyclone, Retsch GmbH, Mettmann, Germany), with a 2 mm mesh used to standardize the particle size. The dry powder was stored in airtight jars inside a desiccator until it was used in the subsequent experimental stages [11,12].

### 2.3. Obtaining the Ethanolic Extract of Propolis

The ethanolic extract of propolis was obtained by dissolving 15 g of raw propolis in 100 mL of 80% ethanol (*v*/*v*) under constant stirring at 1100 rpm for 24 h. The mixture was filtered using a vacuum pump (Model WP6122050, Millipore, MA, USA) equipped with a 0.42 mm filter. The filtrate was centrifuged in a refrigerated centrifuge (TDL-5M, Bioridge, Shanghai, China), adding a few drops of distilled water to facilitate the removal of waxy residues. The clarified extract was concentrated in a rotary evaporator (HS-2005V-N, HAHNVAPOR, Shanghai, China) at 40 °C until its volume was reduced to 20% of the initial volume, promoting the evaporation of ethanol. Finally, the concentrate was stored at 4 °C in amber bottles until it was used. After ethanol evaporation, the extract presented a propolis solids content of 30% *w*/*w*, which served as the basis for formulating the microcapsules [4,6,14].

### 2.4. Microencapsulation by Complex Coacervation

The ethanolic wall material was formulated using a 1:1 (*w*/*w*) ratio of chia mucilage and gelatin, and it was used to prepare coacervate solutions at concentrations of 3%, 4%, and 5% (*w*/*v*). For each treatment, the corresponding amount of biopolymers was weighed and reconstituted in 70 mL of distilled water, with constant stirring at 350 rpm and 40 °C until completely dissolved. Homogenization was performed using an HG-15D mixer (Daihan Scientific, Gangwon, Republic of Korea) at 5000 rpm for 20 s [11,12].

Next, 10 mL of concentrated ethanolic propolis extract was added, and homogenization was repeated at 20 s intervals at 5000 rpm to ensure uniform mixing. The final volume was adjusted to 100 mL with distilled water, maintaining the coacervate concentration defined for each treatment.

Microencapsulation was carried out using a Büchi B-290 spray dryer (Büchi Labortechnik AG, Flawil, Switzerland) equipped with a 0.7 mm double-fluid nozzle. The solutions were processed under drying inlet temperatures of 120 °C, 130 °C, and 140 °C, following a 3^2^ factorial design that evaluated coacervate concentrations of 3%, 4%, and 5%. The following parameters were kept constant: suction at 95%, peristaltic pump at 15%, and stirring at 350 rpm. Drying was completed when the entire volume of each solution had been processed.

Statistical analysis was based on a second-order quadratic model, which describes the effects and interactions of the factors. The equation used was

(1) $$Y=\beta_{0}+\beta_{A}X_{A}+\beta_{B}X_{B}+\beta_{A,B}X_{A}X_{B}+\beta_{A,A}X_{A}^{2}+\beta_{B,B}X_{B}^{2}$$

where Y represents the response variable; $X_{A}$ and $X_{B}$ are the coded variables corresponding to the coacervate concentration and inlet temperature, respectively; $\beta_{0}$ is the constant; $\beta_{A}$ and $\beta_{B}$ are the linear coefficients; $\beta_{A,B}$ is the interaction coefficient; $\beta_{A,A}$ and $\beta_{B,B}$ are the quadratic coefficients of the factors.

### 2.5. Preparation of Ethanolic and Methanolic Extracts for Bioactive Compound Analysis

For raw propolis, 3 g of finely ground sample was mixed with 10 mL of 80% ethanol (*v*/*v*) and subjected to maceration on a magnetic stirrer for 24 h at room temperature. Afterward, the extract was centrifuged and stored for subsequent analysis [15].

For microcapsules and gummy candies, a total of 0.2 g of the sample was weighed into Falcon tubes covered with aluminum foil. Then, 10 mL of 80% methanol (*v*/*v*) was added, and the mixture was homogenized using a vortex at 3000 rpm for 60 s. The samples were kept in the dark at room temperature for 24 h. Subsequently, they were centrifuged in a refrigerated centrifuge (TDL-5M, Bioridge, Shanghai, China) at 3500 rpm for 3 min to separate the solids. The clarified methanolic extract was stored at 4 °C until analysis [4].

### 2.6. Determination of Total Phenolic Compounds

The total phenolic content was determined using the Folin–Ciocalteu method. A standard calibration curve (R^2^ = 0.99) was constructed using gallic acid at concentrations ranging from 1 to 30 mg/L, with the following calibration points: 1, 2, 5, 10, 15, 20, 25, and 30 mg/L. For the analysis, a 0.25 N Folin–Ciocalteu reagent solution and a 20% (*w*/*v*) solution of anhydrous sodium carbonate (Na_2_CO_3_) were prepared. In test tubes covered with aluminum foil, 0.90 mL of methanolic extract was added, followed by 2.4 mL of ultrapure water (dilution factor: 3.67). Then, 0.15 mL of 20% Na_2_CO_3_ and 0.3 mL of Folin–Ciocalteu reagent were added. The mixture was allowed to react for 10 min at room temperature. Finally, absorbance was measured at 755 nm using a UV-Vis spectrophotometer (Genesys 150, Thermo Fisher Scientific, Madison, WI, USA) [16].

The absorbance values were interpolated using the gallic acid calibration curve (y = 0.0379x − 0.0046), where y represents the absorbance and x represents the concentration of phenolic compounds in mg/L. These values were adjusted considering the dilution factor and the sample mass, and the final results were expressed as milligrams of gallic acid equivalents per gram of sample (mg GAE/g); these values were expressed on a dry weight basis, using the moisture content of each sample to perform the conversion.

The encapsulation efficiency (%EE) was calculated under optimal treatment conditions by comparing the total phenolic content in the ethanolic propolis extract with that in the resulting microcapsules. This evaluation allowed for the estimation of the proportion of compounds retained after the complex coacervation encapsulation process [6].

### 2.7. Determination of Flavonoids

The flavonoid content was determined using a standard calibration curve (R^2^ = 0.99) constructed using quercetin as the reference compound at concentrations ranging from 0.2 to 1.2 mg/mL, with the following calibration points: 0.20, 0.40, 0.60, 0.80, 1.00, and 1.20 mg/mL. For the assay, 90.0 µL of methanolic extract, 1.91 mL of 80% ethanol, 100 µL of 5% (*w*/*v*) aluminum chloride (AlCl_3_), and an additional 2.90 mL of 80% ethanol were added in that order. The mixture was then incubated at room temperature in the dark for 30 min. Measurements were taken at a wavelength of 425 nm using a UV-Vis spectrophotometer (Genesys 150, Thermo Fisher Scientific, Madison, WI, USA) [17].

The absorbance values were interpolated using the quercetin calibration curve (y = 1.54499x − 0.0061) to determine the flavonoid concentration in mg/mL. These values were corrected based on the dilution factor and sample mass, and the results were expressed as milligrams of quercetin equivalents per gram of sample (mg QE/g). These values were expressed on a dry weight basis, using the moisture content of each sample to perform the conversion.

### 2.8. Determination of Antioxidant Capacity by the DPPH Method

Trolox was used as the reference compound, and a standard calibration curve was obtained in the range of 20 to 700 µmol/L using the following calibration points: 20, 30, 40, 50, 100, 200, 300, 400, 500, 600, and 700 µmol/L with a determination coefficient of R^2^ = 0.99. The diluted DPPH solution was adjusted to an initial absorbance of 1.10 at 515 nm. For the assay, 150.0 μL of the methanolic extract was mixed with 2.85 mL of the DPPH solution and allowed to react in the dark for 15 min. As a control, a mixture of 150.0 µL of 80% ethanol and 2.85 mL of DPPH solution was used. Absorbance measurements were taken at 515 nm using a UV-Vis spectrophotometer (Genesys 150, Thermo Fisher Scientific, Madison, WI, USA) [4]. The antioxidant capacity of DPPH was expressed as micromoles of Trolox equivalent per gram of sample (µmol TE/g), calculated on a dry weight basis by using the moisture content of each sample to perform the conversion.

### 2.9. Determination of Antioxidant Capacity by the ABTS Method

The ABTS^+^ radical was generated by mixing 250 µL of a 2.45 mM K_2_S_2_O_8_ solution with 25 mL of a 7.00 mM ABTS solution and allowing the mixture to stand in the dark for 8 h. For the assay, 300 μL of the methanolic extract was combined with 2.7 mL of the ABTS^+^ solution. Absorbance was measured at 734 nm using a UV-Vis spectrophotometer (Genesys 150, Thermo Fisher Scientific, Madison, WI, USA). Trolox was used as the reference compound for the standard curve, which was constructed using concentrations of 10, 50, 100, 200, and 300 µmol/L. The resulting calibration curve was described by the equation y = 0.0017x + 0.1306, with a determination coefficient of R^2^ = 0.98 [4]. The antioxidant capacity of ABTS was expressed as µmol TE/g on a dry weight basis, using the moisture content of each sample to perform the conversion.

### 2.10. Color Determination

Color analysis was performed using a CR-5 benchtop colorimeter (Konica Minolta, Tokyo, Japan), employing a 40.5 × 60.0 mm cell and the reflectance accessory. The results were expressed in the CIELAB system (L*, a*, and b*) [18].

### 2.11. Moisture Determination

The moisture content was determined using the official AOAC method 950.10. A total of 0.2 g of sample was weighed and dried in a forced convection oven (FED 115, BINDER GmbH, Tuttlingen, Germany) at 105 °C until a constant weight was achieved [18].

### 2.12. Hygroscopicity Determination

A total of 0.2 g of sample was weighed and placed in a sealed container with a saturated NaCl solution, maintaining controlled conditions for 7 days. After this period, the final mass was recorded, and hygroscopicity was calculated using the following formula:

(2) $$I=\left(\frac{M_{3}-M_{2}}{M_{2}-M_{1}}\right)\times 100$$

where I is the percentage increase in mass; $M_{1}$ is the mass of the empty Petri dish; $M_{2}$ is the mass of the Petri dish with the sample at the beginning; and $M_{3}$ is the mass of the Petri dish with the sample after seven days [17].

### 2.13. Bulk Density Determination

A known amount of dry, unpacked sample was placed in a 10.0 mL graduated cylinder, and the volume occupied was recorded. The mass was then measured using an analytical balance. Bulk density was calculated as the ratio of the sample mass to the displaced volume and expressed in g/mL [4].

### 2.14. Solubility Determination

Solubility was quantified by dissolving 0.5 g of microcapsules in 20 mL of distilled water. The mixture was vortexed at 3000 rpm for 2 min and then incubated in a water bath at 80 °C for 30 min. After this period, the sample was allowed to cool to room temperature and then centrifuged at 3500 rpm for 10 min. The supernatant was transferred to a Petri dish and dried in an oven at 90 °C for 24 h. Solubility was calculated using the following formula:

(3) $$S=\left(\frac{M_{2}}{M_{1}}\right)\times 100$$

where S is the solubility percentage; $M_{1}$ is the initial mass of the microcapsules (g); and $M_{2}$ is the mass of the dry residue obtained from the supernatant (g) [4].

### 2.15. Particle Size Determination

Particle size was determined by laser diffraction using a Mastersizer 3000 instrument (Malvern Instruments, Worcestershire, UK). A total of 0.02 g of microcapsules was dissolved in 50 mL of isopropyl alcohol, and the mixture was stirred at 3000 rpm for 2 min. It was then subjected to ultrasound in a DU-220S ultrasonic bath (ARGO LAB, Milan, Italy) for 10 min at 40 kHz. Measurements were taken at a wavelength of 600 nm. The polydispersity index (PDI) was calculated using the following equation:

(4) $$PDI=\frac{D_{\left(0.9\right)}-D_{\left(0.1\right)}}{D_{\left(0.5\right)}}$$

where $D_{\left(0.1\right)}$, $D_{\left(0.5\right)}$, and $D_{\left(0.9\right)}$ are the diameters corresponding to 10%, 50%, and 90% of the cumulative particle size distribution, respectively [19].

### 2.16. ζ Potential Determination

ζ potential was determined by dynamic light scattering (DLS) using a Zetasizer ZU3100 (Malvern Instruments, Worcestershire, UK). A total of 0.02 g of sample was dissolved in the appropriate medium and subjected to ultrasound for 10 min at 40 kHz. Measurements were performed in a DTS1070 cell under the instrument’s standard conditions [19].

### 2.17. In Vitro Release Evaluation of Phenolic Compounds

The release was evaluated using the Folin–Ciocalteu method. A microcapsule dispersion at a concentration of 10 mg/mL was prepared and incubated at 25 °C under static conditions for 6, 12, 18, 24, 30, 36, 42, and 48 h. At the end of each interval, the phenolic compound content was determined by spectrophotometric reading at 755 nm using a UV-Vis spectrophotometer (Genesys 150, Thermo Fisher Scientific, Madison, WI, USA) [16].

### 2.18. Scanning Electron Microscopy (SEM) Analysis

The morphology of the microcapsules was evaluated by scanning electron microscopy (SEM) using a Prisma E microscope (Thermo Fisher Scientific, Brno, Czech Republic). The samples were mounted on aluminum stubs using 12 mm carbon adhesive tape. Observations were carried out under low-vacuum conditions using ABS and LVD detectors at a pressure of 0.07 Torr [6].

### 2.19. Fourier Transform Infrared (FTIR) Spectroscopy Analysis

Functional group analysis was performed by FTIR spectroscopy in transmission mode using a Nicolet iS50 spectrophotometer (Thermo Fisher Scientific, Waltham, MA, USA). Pellets were prepared by pressing a mixture of 200 mg of KBr and 2 mg of sample under 10 tons of pressure for 40 s. Spectra were acquired in the mid-infrared range using a KBr beam splitter, with a resolution of 8 cm^−1^ and 32 scans [6].

### 2.20. Thermogravimetric Analysis (TGA)

Functional thermogravimetric analysis was performed using a TGA 550 instrument (TA Instruments, New Castle, DE, USA). A total of 10 mg of microcapsules was heated from room temperature to 800 °C at a heating rate of 10 °C/min under a nitrogen atmosphere [16].

### 2.21. Differential Scanning Calorimetry (DSC)

Differential scanning calorimetry analysis was performed using a DSC 2500 instrument (TA Instruments, New Castle, DE, USA). A total of 2 mg of microcapsules was used, applying a heating program from 20 °C to 300 °C at a rate of 3 °C/min [16].

### 2.22. Ultra-High-Performance Liquid Chromatography Analysis (UPLC-PDA-QDa)

The microcapsules from the optimal treatment were obtained from ethanolic propolis extract previously concentrated by rotary evaporation, followed by a complex coacervation process using chia mucilage and gelatin, and finally spray-dried. The identification and quantification of phenolic compounds were carried out using external calibration curves specific to each analytical standard. Analyses were performed in triplicate, and the results were expressed as mean ± standard deviation in mg/g of dry sample.

An ultra-high performance liquid chromatography (UPLC) system coupled to a photodiode array detector (PDA) and a single quadrupole mass detector (QDa) (ACQUITY UPLC-PDA-QDa, Waters Corporation, Milford, MA, USA) was used. The separation was performed on a C18 column (2.1 × 100 mm, 1.8 µm) maintained at 40 °C, using a mobile phase consisting of water (94.8%) with 80% methanol (5%) and formic acid (0.2%) (phase A), and acetonitrile with 0.2% formic acid (phase B). The elution gradient was programmed from 5% to 85% of phase B over 11.2 min, with a flow rate of 0.5 mL/min and an injection volume of 5.0 µL.

Detection was performed at 254 nm with the PDA and in both positive (ESI^+^) and negative (ESI^−^) electrospray ionization modes with the QDa within an *m*/*z* range of 100 to 600. Compound identification was based on the comparison of retention times, UV spectra, and exact *m*/*z* values with those of commercial analytical standards. Limits of detection (LODs) and quantification (LOQs) were determined for each compound to ensure the reliability and sensitivity of the quantification results. All identified compounds matched the reference data of the corresponding standards used [20,21,22].

### 2.23. Incorporation into Gummy Candies

Microcapsules obtained by complex coacervation were incorporated into three formulations of functional gummy candies using different concentrations of microencapsulated material and red cactus pear juice, as shown in Table 1. The mixture was prepared at 65 °C with constant stirring until a homogeneous mass was obtained. It was then poured into silicone molds and allowed to set at room temperature until solidified, followed by storage at 18 °C [23].

### 2.24. Sensory Evaluation

Sensory evaluation was conducted two weeks after storage of the gummy candies enriched with propolis microcapsules, which were obtained by complex coacervation. Portions of 20 g were offered to 70 untrained panelists, aged between 16 and 22 years, selected by convenience sampling, ensuring a balanced gender distribution (50% female and 50% male), and with no prior experience with functional products.

Each sample was assigned a random three-digit code to ensure unbiased evaluation. The tests were conducted in a well-lit environment, free from interfering odors and with adequate ventilation.

The study included two consumer-type tests [23]:

A preference test, in which each participant selected the formulation they liked the most.

An acceptability test using a five-point structured hedonic scale, where 1 = dislike very much, 2 = dislike, 3 = neither like nor dislike, 4 = like, and 5 = like very much. The attributes evaluated were color, aroma, flavor, and texture.

The panel size was determined using Cochran’s formula for proportions, considering a 95% confidence level and a 10% margin of error, which validated that those 70 participants constituted a representative sample size. All participants provided informed consent, and the Ethics Committee of the National University José María Arguedas approved the test.

### 2.25. Texture Profile Analysis (TPA)

Texture profile analysis of the gummy candies was performed using a Brookfield AMETEK Texture Analyzer equipped with a 5000 g load cell. Samples were subjected to a compression test using a cylindrical TA3/1000 probe with 40% deformation at a speed of 1 mm/s. A TPA test with two compression cycles was used to evaluate hardness, cohesiveness, adhesiveness, springiness, and resilience. For each formulation (F1, F2, and F3), three replicates were analyzed, and average values with standard deviations were obtained. Data analysis was performed using Texture Pro software version 1.0.19.

### 2.26. Statistical Analysis

Physicochemical and instrumental properties were analyzed using ANOVA and Tukey’s multiple comparison test, with a significance level of 5% and three replicates per treatment. For the sensory data, the Kolmogorov–Smirnov normality test was applied first, followed by the non-parametric Friedman and Wilcoxon tests. In the microencapsulation study, a 3^2^ factorial design was employed, evaluated using a quadratic model, and optimized through the desirability function. Statistical analysis was performed using Design Expert v9.0 software (Trial version, Stat-Ease Inc., Minneapolis, MN, USA) and Origin Pro 2025 software (OriginLab Corporation, Northampton, MA, USA).

## 3. Results and Discussions

### 3.1. Characterization of Raw Propolis and Ethanolic Propolis Extract

Table 2 presents the physicochemical properties of raw propolis and its ethanolic extract, showing significant differences between them. The results demonstrated that the extraction process increased the phenolic compounds (17.66 mg gallic acid equivalents (GAE)/g), flavonoids (13.72 mg quercetin equivalents (QE)/g), and antioxidant capacity by DPPH (102.88 µmol Trolox equivalents (TE)/g). Additionally, the extract exhibited higher lightness (L*) and greater values of chroma (a* and b*). A higher L* indicates a lighter color, while increases in a* and b* reflect greater intensity in red and yellow tones. These results demonstrate a more vivid and saturated color in the ethanolic extract compared to the raw propolis. These changes are attributed to the ethanolic extraction process, which removes waxes and other non-functional fractions present in raw propolis; this technique facilitates the concentration of bioactive metabolites responsible for the higher antioxidant capacity of the extract [4,24].

### 3.2. Characterization of Wall Materials

**Figure 1:**
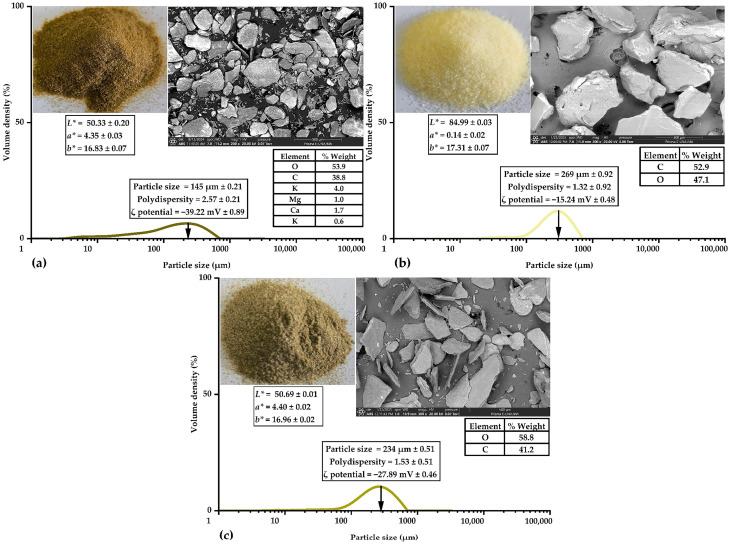
Characterization of wall materials: chia mucilage (**a**), gelatin (**b**), and 1:1 mixture of chia mucilage and gelatin (**c**).

Figure 1 shows the physicochemical and structural properties of the studied wall materials. First, chia mucilage (Figure 1a) exhibited smaller particle size, lower lightness, and higher polydispersity, indicating a more heterogeneous distribution. Second, gelatin (Figure 1b) exhibited a larger particle size, higher lightness, and lower polydispersity, indicating a more homogeneous distribution. Finally, the 1:1 mixture of chia mucilage and gelatin (Figure 1c) presented intermediate values of particle size, lightness, and polydispersity, resulting in an encapsulating matrix with a favorable structural balance for conducting the complex coacervation process.

Moreover, ζ potential values indicated varying colloidal stability, which was higher in the case of chia mucilage. Additionally, SEM-EDS analysis revealed differences in particle morphology and elemental composition, characteristic of naturally derived polysaccharides. These findings are consistent with previous studies that highlight the complementary behavior of chia mucilage and gelatin in complex coacervation systems, where their structural and colloidal properties promote the formation of stable and functional capsules [11,12,13].

### 3.3. Physicochemical Properties of Microcapsules Obtained by Complex Coacervation and Spray Drying

Table 3 presents the results of the 3^2^ factorial design, which evaluated the effect of the coacervate concentration (3%, 4%, and 5%) and inlet temperature (120 °C, 130 °C, and 140 °C) on the physicochemical properties of the obtained microcapsules. The results showed wide ranges for the evaluated dependent variables, including phenolic compounds (9.16–15.36 mg GAE/g), flavonoids (2.67–7.77 mg QE/g), antioxidant capacity measured by DPPH (36.78–60.10 µmol TE/g) and ABTS (22.19–50.10 µmol TE/g), solubility (41.49–79.72%), hygroscopicity (12.58–20.50%), moisture content (2.70–9.57%), bulk density (177.75–284.25 g/cm^3^), particle size (5.39–7.80 µm), and ζ potential (−37.29 to −13.20 mV). These results demonstrate that the operating conditions during spray drying significantly affect the structural and functional stability of the microcapsules, providing a valuable framework for their optimization [13,17].

Table 4 presents the results of the analysis of variance (ANOVA) for the response variables of the factorial design. The experimental data showed a significant fit to the predicted mathematical models, with coefficients of determination (R^2^) ranging from 0.51 to 0.97. These results allowed the experimental values to be fitted to the proposed mathematical model, considering the R^2^ values and statistical significance (*p* ≤ 0.05) to optimize the conditions of the spray-drying microencapsulation process [17,25].

**Figure 2:**
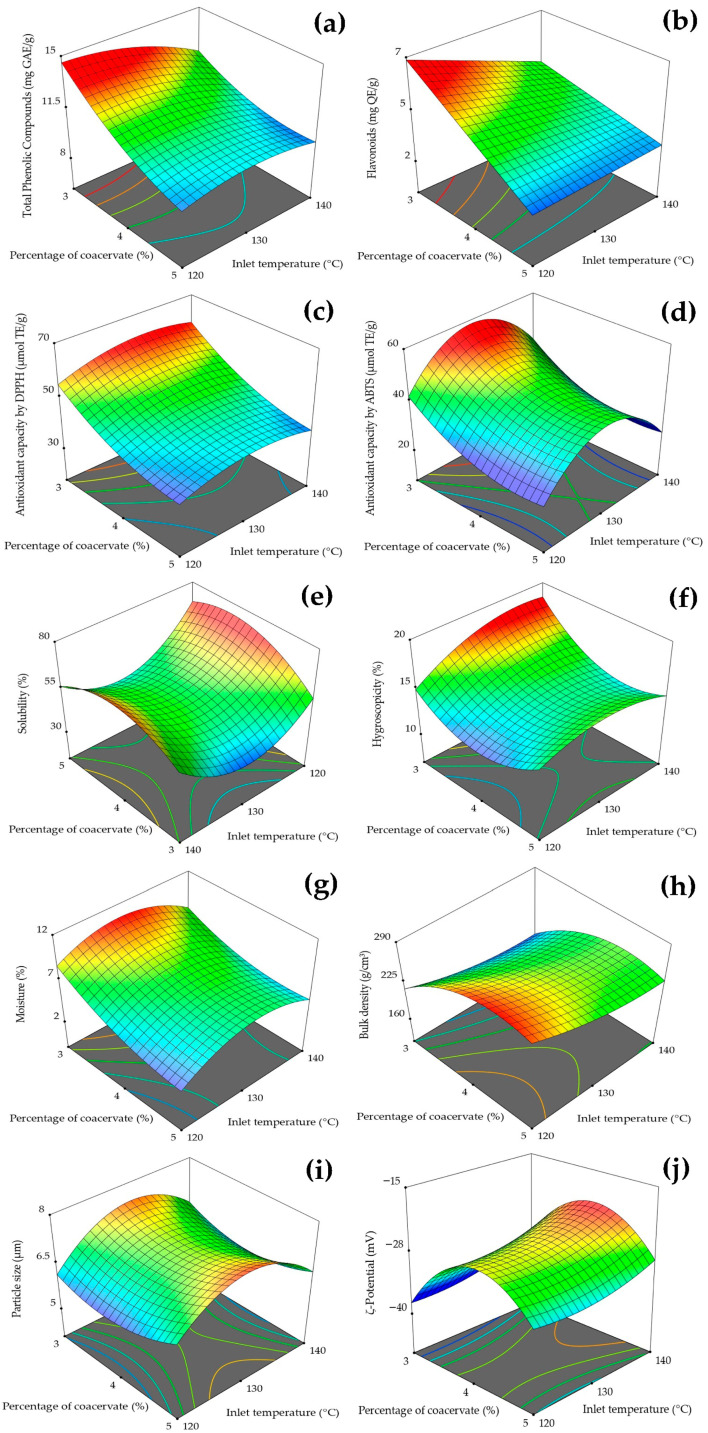
Response surface plots showing the effect of coacervate concentration and inlet temperature on: (**a**) total phenolic compounds, (**b**) flavonoids, (**c**) antioxidant capacity by DPPH, (**d**) antioxidant capacity by ABTS, (**e**) solubility, (**f**) hygroscopicity, (**g**) moisture, (**h**) bulk density, (**i**) particle size, and (**j**) ζ-potential.

The response surface plots shown in Figure 2 illustrate the influence of coacervate concentration (A) and inlet temperature (B) on the properties of the microcapsules. A significant fit was observed between the mathematical models and the experimental data, indicating good predictive capacity for most of the evaluated variables.

Phenolic compounds (Figure 2a) showed high statistical significance (*p*-value ≤ 0.0001) and a satisfactory model fit (R^2^ = 0.85). Their retention was mainly influenced by the linear effects of A and B, the quadratic effect of A, and the interaction of AB. In the case of flavonoids (Figure 2b), the model also revealed a significant relationship (*p*-value ≤ 0.0001, R^2^ = 0.79), with the linear effects of A and B, and the interaction AB, being significant. Regarding antioxidant capacity measured by DPPH (Figure 2c), a strong correlation was found (*p*-value ≤ 0.0001, R^2^ = 0.95), with significant linear effects of A and B, and significant quadratic effects of A and B. In the case of the antioxidant capacity determined by ABTS (Figure 2d), a strong correlation was also observed (*p*-value ≤ 0.0001, R^2^ = 0.86), with a significant linear effect of A and significant quadratic effects of both A and B. Previous studies have shown that moderate inlet temperatures and adequate encapsulant ratios enhance the stability of bioactive compounds by reducing thermal degradation [4,6]. These findings emphasize the importance of avoiding high temperatures during encapsulation, as the wall material serves as a protective barrier against oxidation and heat, thereby preserving the antioxidant activity [17,25,26,27,28].

The physical properties were also significantly affected by the process parameters. Solubility (Figure 2e) showed a significant relationship with the model (*p*-value ≤ 0.0001, R^2^ = 0.87), with significant effects from the linear terms, interaction, and quadratic effects of A and B. For hygroscopicity (Figure 2f), a moderately significant correlation was observed (*p*-value ≤ 0.008, R^2^ = 0.51), with both the linear and quadratic effects of A being influential. Moisture content (Figure 2g) showed a strong, significant correlation (*p*-value ≤ 0.0001, R^2^ = 0.75), influenced by the linear effect of A and the quadratic effect of B. On the other hand, bulk density (Figure 2h) showed a highly significant correlation (*p*-value ≤ 0.0001, R^2^ = 0.97), with significant contributions from the linear and quadratic effects of both A and B. For particle size (Figure 2i), a moderate correlation was found (*p*-value ≤ 0.0003, R^2^ = 0.65), where the quadratic effects of A and B were relevant. Finally, ζ potential (Figure 2j) showed a moderately significant correlation (*p*-value ≤ 0.0032, R^2^ = 0.55), with a significant effect from the quadratic term of A.

The aforementioned results demonstrate an overall improvement in powder quality under controlled spray-drying conditions, with positive effects also observed on the stability and functionality of the microcapsules. Controlling temperature and encapsulant concentration is thus key to optimizing the final product [4,25,29,30,31]. Unlike conventional encapsulating systems such as maltodextrin or gum Arabic, the combination of chia mucilage and gelatin offers additional functional advantages. This mixture provides greater gelling capacity, contributes soluble fiber, and may exert prebiotic effects. Furthermore, the mucilage acts as a natural stabilizer, reducing the thermal degradation of phenolic compounds during drying. This structural synergy could explain the high phenolic retention and sustained antioxidant activity observed in the propolis microcapsules [11,12].

To determine the optimal experimental conditions, a multiple-response analysis based on the desirability function was applied, with values ranging from 0 to 1 [32]. Table 5 shows that the optimal treatment was achieved with a 3.13% coacervate concentration and an inlet temperature of 120 °C, resulting in an overall desirability of 0.59. This result was obtained by maximizing the values of phenolic compounds, flavonoids, antioxidant capacity, and solubility while minimizing the values of hygroscopicity, moisture content, bulk density, particle size, and zeta potential.

The experimental results for the optimal treatment fell within the 95% confidence interval. They achieved prediction accuracy values above 90%, indicating the robustness of the factorial design used and the importance of adjusting process conditions to enhance the functionality of propolis microcapsules for potential applications in the food, pharmaceutical, and cosmetic industries.

The encapsulation efficiency (%EE) of phenolic compounds under optimal treatment was 80.75 ± 0.01%. This result demonstrates the effectiveness of the complex coacervation process, using chia mucilage and gelatin, in retaining bioactive compounds. This value is attributed to the moderate drying conditions and the appropriate proportion of biopolymers used, which helped minimize thermal degradation [6,12,21].

### 3.4. Identification and Quantification of Phenolic Compounds in the Optimal Treatment

Table 6 presents the phenolic compounds identified in the microcapsules obtained under optimal treatment conditions. Identification was performed by comparing retention times, molecular masses, and spectra with analytical standards. Nine phenolic compounds were identified: gallic acid, catechin, epicatechin, epigallocatechin gallate, rutin, myricetin, resveratrol, quercetin, and kaempferol. Retention times ranged from 0.76 to 9.12 min, with molecular weights from 170.12 to 610.52 g/mol. Catechin and epicatechin showed the highest concentrations, at 2.303 and 2.275 mg/g, respectively. Meanwhile, quercetin was detected at the lowest concentration (0.005 mg/g), possibly due to its high susceptibility to thermal degradation [21]. The presence of catechin, epicatechin, and resveratrol in these samples reflects the botanical variability of propolis from this Andean region (Andahuaylas), influenced by local species such as *Eucalyptus globulus* (eucalyptus), *Escallonia myrtilloides* (tasta), *Baccharis lanceolata* (chilca), *Prunus serotina* (capulí), *Alnus jorullensis* (alder), *Cupressus macrocarpa* (cypress), *Brassica napus* (wild mustard), and *Pinus radiata* (pine) [4]. Moreover, the use of ethanol as an extraction solvent favors the solubilization of flavanols and stilbenes, as previously reported in studies involving ethanolic extracts of propolis [33,34,35]. Likewise, the encapsulation system using complex coacervation with chia mucilage and gelatin favored the stabilization and recovery of the bioactive compounds during spray drying [10].

**Figure 3:**
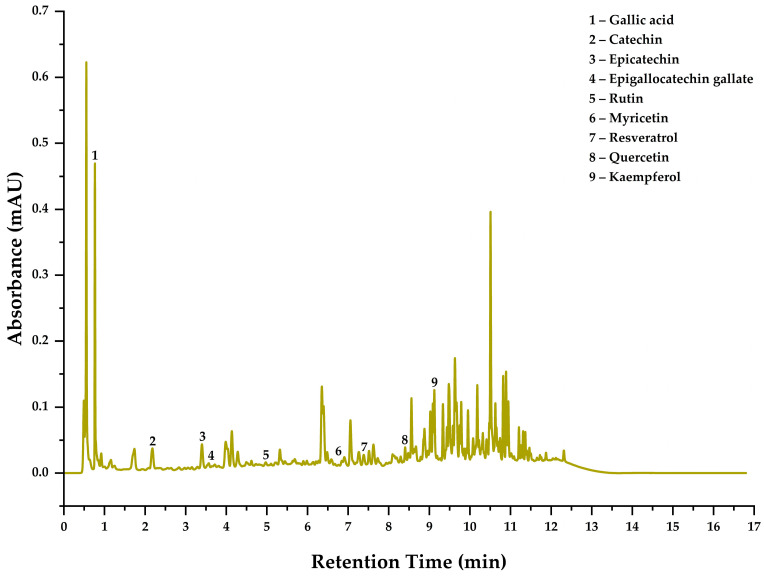
Chromatogram of the phenolic compounds identified in the microcapsules.

The corresponding chromatogram is shown in Figure 3, where the numbered peaks correlate with the phenolic compounds listed in Table 6. The detected compounds belong to the classes of phenolic acids, flavanols, flavonols, and stilbenes, all of which are recognized for their antioxidant, anti-inflammatory, and cardioprotective properties [20,21]. Rutin stands out due to its high molecular weight (610.52 g/mol) and glycosylated structure, making it particularly susceptible to thermal and oxidative degradation. Its clear detection after spray drying confirms the protective effect of the microencapsulation system [21].

The identified compounds (gallic acid, epicatechin, rutin, resveratrol, quercetin, and kaempferol) are consistent with those reported by Duca et al. (2019) in multi-floral propolis from Western Romania, where resveratrol was detected in all analyzed samples, reaching up to 19.77% of total polyphenols [33]. Similarly, Volpi (2004) identified resveratrol, catechin, quercetin, rutin, and kaempferol in commercial propolis extracts from Italy, demonstrating that these compounds are also present in propolis from different regions and extraction matrices [34]. A global review on the chemical composition of propolis conducted by Huang et al. (2014) reported the detection of prenylated stilbenes, such as 5-farnesyl-3′-hydroxyresveratrol, 4-prenyldihydroresveratrol, and 3-prenylresveratrol, in propolis samples collected from Australia, Brazil, Greece, Indonesia, and Kenya, attributing their botanical origin to local plant species used by *Apis mellifera* [36].

In addition, Rocha et al. (2024) [35] identified gallic acid, epicatechin, rutin, resveratrol, quercetin, and kaempferol in stingless bee propolis from Brazil, confirming the recurrence of these key metabolites across different regions and bee species. These findings demonstrate that the phenolic profile is strongly influenced by the botanical environment and bee biology, granting unique characteristics to the encapsulated propolis [4]. The detection of resveratrol is particularly relevant, as it suggests the influence of the local flora, especially *Eucalyptus globulus*, as a probable botanical source. This compound is found in a wide range of plants, including species of the *Myrtaceae* family, of which eucalyptus is a prominent representative and a floral source frequently visited by bees in various ecosystems [4,33,35,37].

Altogether, the simultaneous detection of these compounds after the encapsulation and spray-drying process confirms the effectiveness of this technique in preserving sensitive functional metabolites, particularly those with high reactivity and bioactive value, such as resveratrol and rutin [22].

### 3.5. Morphological, Structural, and Thermal Evaluation of the Optimal Treatment

**Figure 4:**
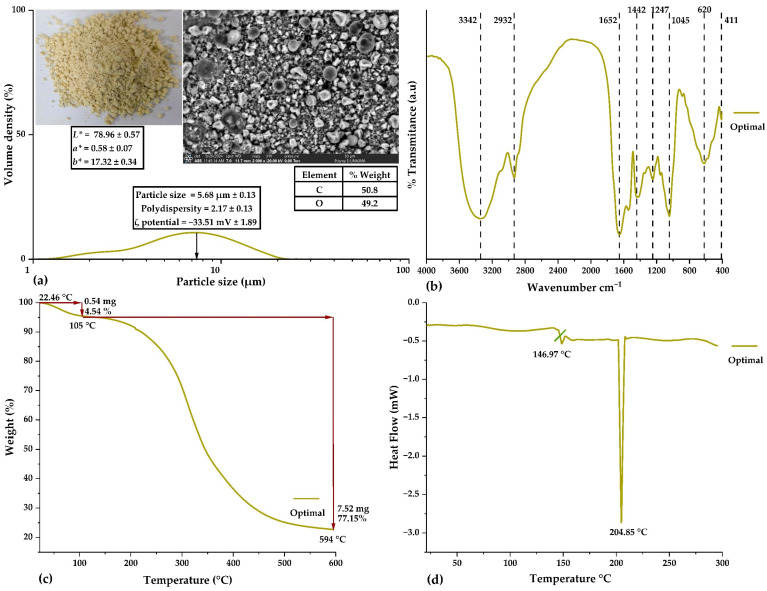
Physical, chemical, and structural properties of the optimal treatment: (**a**) particle size distribution, color, ζ potential, and micrograph (SEM and EDS); (**b**) FTIR spectrum; (**c**) thermogravimetric analysis (TGA); (**d**) differential scanning calorimetry (DSC).

Figure 4 shows the morphological, structural, and thermal characterization of the microcapsules obtained under optimal conditions. These analyses enabled the identification of key properties that support the effectiveness of the microencapsulation process.

Figure 4a displays the particle size distribution, with an average value of 5.68 µm and a polydispersity index of 2.17, indicating a relatively uniform distribution suitable for food, pharmaceutical, and cosmetic applications [6,38]. The ζ potential analysis yielded a value of −33.51 mV, indicating good colloidal stability, which is essential for preventing agglomeration during storage and enhancing the solubility of the final product [4,27]. In addition, color analysis revealed that the chromatic coordinates resulted in a light and homogeneous color associated with the stability of the bioactive compounds encapsulated under optimal conditions [4,6]. The SEM micrograph showed a spherical morphology with continuous and well-defined surfaces. Complementary EDS analysis confirmed that the predominant elemental composition was carbon (50.8%) and oxygen (49.2%), consistent with the organic nature of propolis and the biopolymers used as wall materials [4,6].

In Figure 4b, the FTIR spectrum confirms the presence of physical interactions between propolis and the wall materials, with no evidence of chemical reactions. Characteristic peaks were observed at 3342 cm^−1^ and 2932 cm^−1^, associated with hydroxyl groups, amino acids, and carboxylic acids; the peak at 1652 cm^−1^ corresponds to phenolic compounds and flavonoids, while the one at 1045 cm^−1^ indicates the presence of ether and alcohol groups. Other minor peaks are related to aromatic rings and possible modifications induced by spray drying [4,6]. These results confirm that microencapsulation is a physical process that preserves the stability of bioactive compounds, validating their structural integrity for industrial applications [39,40].

Figure 4c, corresponding to the TGA, shows an initial weight loss of 4.54% at 105 °C, attributed to the removal of moisture. Continuous thermal degradation begins at 594 °C, indicating a significant improvement in the thermal stability of encapsulated propolis compared to its free form [4,6].

Finally, Figure 4d, corresponding to the DSC analysis, shows an endothermic transition at 146.97 °C, associated with microcapsule dehydration, and a main transition at 204.85 °C, indicating the thermal stability of the encapsulating matrix under controlled conditions [16,19].

Taken together, these results demonstrate the effectiveness of the microencapsulation process in enhancing the thermal, colloidal, and structural stability of propolis, consolidating this technique as a promising strategy for its application in the food, pharmaceutical, and cosmetic industries.

### 3.6. In Vitro Release of Phenolic Compounds

**Figure 5:**
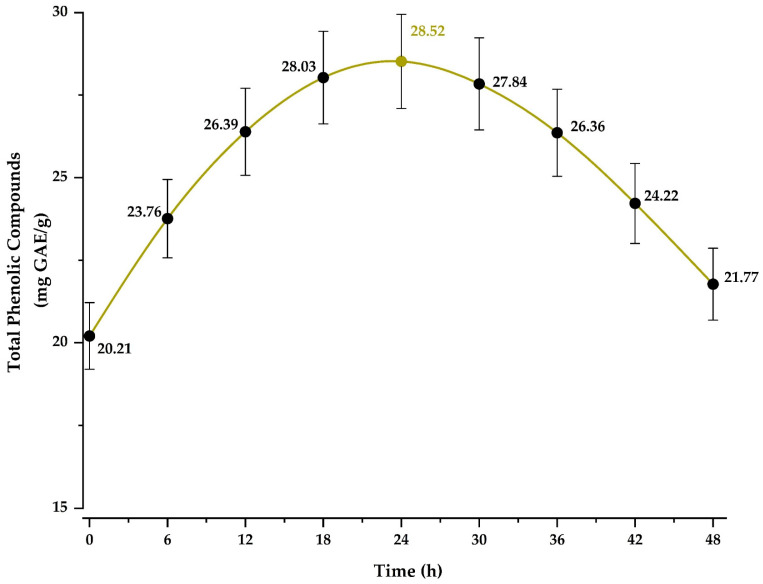
Release of total phenolic compounds in the optimal treatment.

Figure 5 shows the release profile of phenolic compounds in the optimal treatment, with a maximum concentration of 28.52 mg GAE/g reached at 24 h. Afterward, a gradual decline in the released concentration was observed, reaching 21.77 mg GAE/g at 48 h. This behavior demonstrates the encapsulating matrix’s ability to control the release of bioactive compounds and protect them from degradation, thereby ensuring sustained delivery. These findings highlight the potential of the encapsulation system for use in functional products with controlled release in the pharmaceutical, cosmetic, and food industries [16,17,41].

The observed controlled release may be attributed to the semi-structured network formed through complex coacervation between chia mucilage and gelatin, whose electrostatic interaction created a diffusive barrier that modulated the release of phenolic compounds [11,12].

### 3.7. Incorporation of Microcapsules into Functional Gummy Candies

**Figure 6:**
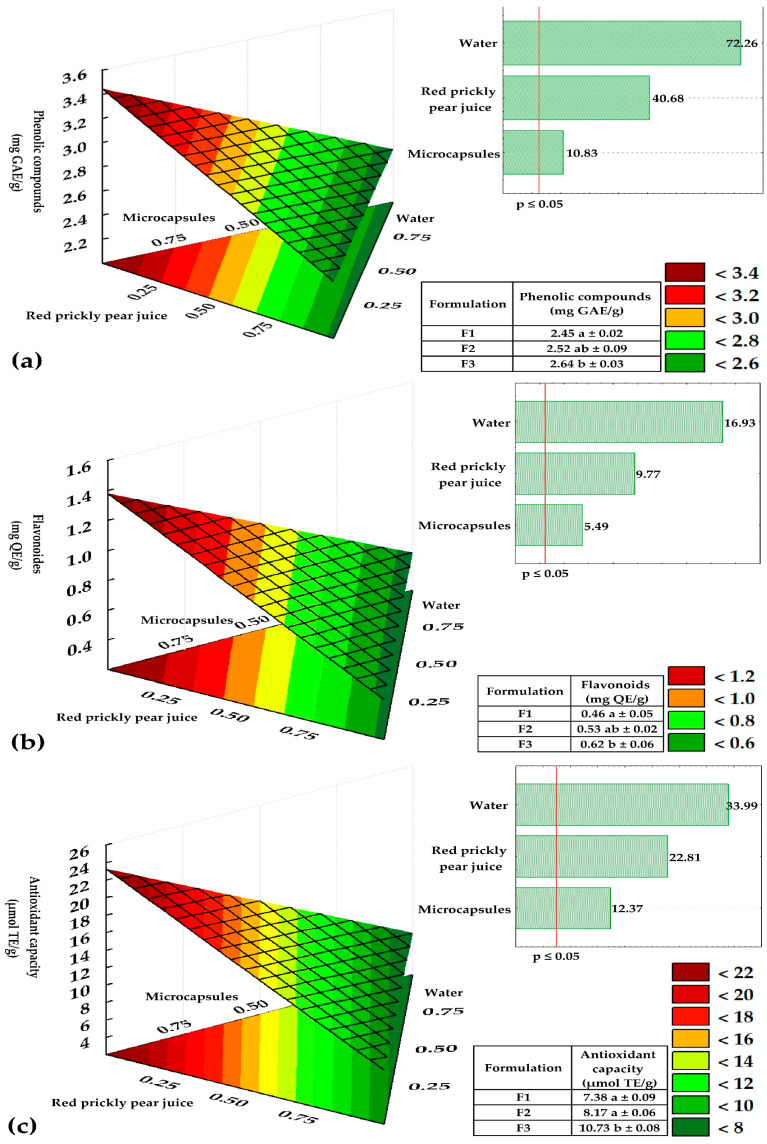
Functional properties of gummy candies: (**a**) phenolic compounds, (**b**) flavonoids, and (**c**) antioxidant capacity. Different letters (a, b) within the same column in the tables indicate statistically significant differences (*p* ≤ 0.05) according to Tukey’s test.

Figure 6 shows the effect of adding propolis microcapsules obtained from the optimal treatment on the functional properties of gummy candies. It was observed that formulation F3, with the highest content of microcapsules and red cactus pear juice, reached the highest levels of phenolic compounds (2.64 mg GAE/g, Figure 6a), flavonoids (0.62 mg QE/g, Figure 6b), and antioxidant capacity by DPPH (10.73 µmol TE/g, Figure 6c). Conversely, a higher percentage of water significantly reduced these values, indicating a dilution effect on the bioactive compounds. The Pareto charts and response surface plots confirm that the optimal combination of microcapsules and red cactus pear juice significantly (*p* ≤ 0.05) enhances the functional properties of the gummy candies. In contrast, a higher proportion of water negatively affects their functionality.

The phenolic compound levels exceeded those reported by Cedeño et al. [42], who obtained values ranging from 0.165 to 0.271 mg GAE/g in gelatin candies enriched with green propolis. Similarly, the antioxidant capacity was higher than that reported by the same authors (0.55 to 1.35 µmol TE/g). Additionally, the phenolic compound levels were also higher than those reported by Kaewpetch et al. [38], who developed honey-based gummies with propolis extract (*Apis cerana*), reaching a maximum of 2.13 mg GAE/g.

**Figure 7:**
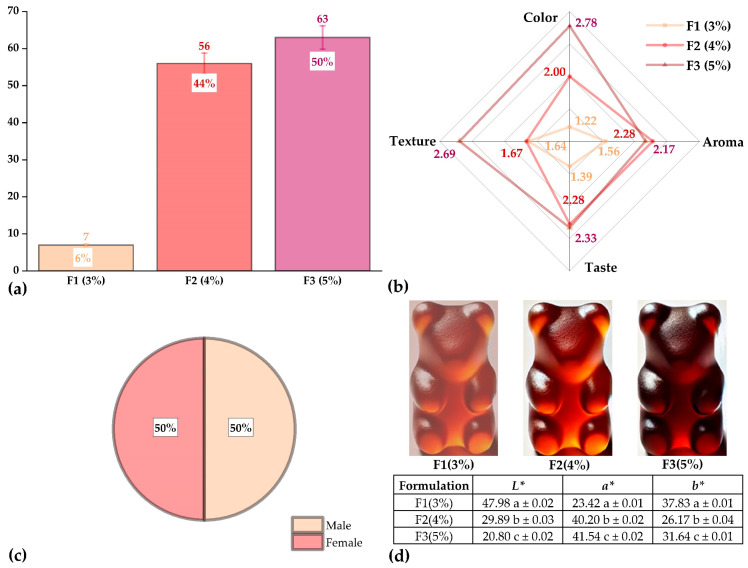
Characterization of gummy candies: (**a**) preference test, (**b**) acceptability test, (**c**) gender distribution, and (**d**) instrumental color of the gummies. L*, a*, and b* represent the CIELAB color space parameters. Different letters (a, b, c) within the same column indicate statistically significant differences (*p* ≤ 0.05) according to Tukey’s test.

Figure 7 shows the sensory evaluation and instrumental color characterization of the functional gummy candies containing propolis microcapsules.

Figure 7a presents the results of the preference test, where formulation F3 (50%) was the most preferred by the panelists. Figure 7b shows the attribute-based sensory analysis (color, aroma, texture, and flavor), where F3 also received the highest scores in most categories, except for aroma. The panelists’ preference for F3 in both suggests a more favorable overall sensory perception. This trend may be attributed to the encapsulation process, which could have helped mask undesirable flavors and improve texture, thereby enhancing product acceptance [43,44].

Figure 7c illustrates a balanced gender distribution among panelists, ensuring a well-balanced sensory analysis.

Figure 7d presents the instrumental color values, where a progressive decrease in lightness (L*) is observed with increasing microcapsule content, indicating a darkening of the product. The a* value increased in F3, reflecting a more reddish hue, while the b* value was higher in F1, corresponding to greater yellow intensity. These changes reflect the influence of propolis compounds on the final color.

Similar results were reported by Cedeño et al. [42] and Kaewpetch et al. [38], who also found that higher propolis content negatively affected the aroma attribute but improved color perception. Moreover, both studies reported decreases in L* and variations in a* and b*, which is consistent with our findings and confirms the visual impact of propolis in gel-based matrices.

Table 7 presents the texture profile results of the functional gummy candies. Formulation F3 showed the highest hardness (2.58 N), attributed to its lower water content and higher proportion of microcapsules, whereas F2 recorded the lowest hardness (1.41 N), reflecting a less compact matrix. Regarding cohesiveness, adhesiveness, and springiness, F2 reached the highest values, likely due to the plasticizing effect of water. Similarly, resilience was greater in F2, indicating a better ability of the gel to recover its shape after deformation. In contrast, F3 showed the lowest values in all these parameters, which may be associated with the influence of microencapsulated propolis, which limits the gel’s flexibility and recovery.

These results are consistent with those reported by Cedeño et al. [42] and Kaewpetch et al. [38], who found that incorporating propolis into gelatin-based products and gummy candies can significantly alter the texture, either reducing hardness or increasing stickiness, depending on the concentration and matrix. Moreover, the results showed lower hardness compared to the gummy candies developed by González et al. [45], who reported values ranging from 25.79 to 39.30 N. This behavior is possibly due to the higher water content and the interaction of bioactive compounds with the gelling matrix.

Although the results obtained are promising, this study focused on the in vitro evaluation of antioxidant functionality. It did not include simulated gastrointestinal release assays, which would provide deeper insights into the bioavailability of the encapsulated compounds. Additionally, the formulation was applied only in a gummy candy matrix; therefore, future research could evaluate its behavior in other functional food systems, considering key factors such as storage stability and effective consumer dosage.

## 4. Conclusions

The microencapsulation of propolis by complex coacervation using chia mucilage and gelatin proved to be an effective strategy for protecting and stabilizing phenolic compounds in a gummy candy matrix. The optimal formulation exhibited high levels of total phenolics, flavonoids, and antioxidant capacity, along with favorable sensory acceptance. Liquid chromatography analysis confirmed the presence of bioactive compounds, including gallic acid, catechin, epicatechin, epigallocatechin gallate, rutin, myricetin, resveratrol, quercetin, and kaempferol, which remained detectable even after spray drying, demonstrating the efficiency of the encapsulation system. Overall, the results support the application of this technology in developing functional foods with enhanced stability and potential health benefits. It is worth noting that the use of Generally Recognized As Safe (GRAS) solvents, in combination with emerging extraction techniques, is increasingly promoted as a more sustainable approach to obtaining bioactive compounds. This avenue will be further explored in future studies. Additionally, future research should broaden the panel of analytical standards to achieve a more comprehensive characterization of the phenolic profile.

## Figures and Tables

**Table 1:** Formulation of functional gummy candies.

Formulation	Microcapsules	Red Cactus Pear Juice	Water	Neutral Gelatin (200° Bloom)	Sucrose	Glucose	Isomaltose	Potassium Benzoate	Glycerin
F1	3.00%	35.00%	16.50%	11.00%	11.00%	17.00%	5.00%	1.00%	0.50%
F2	4.00%	30.00%	20.50%	11.00%	11.00%	17.00%	5.00%	1.00%	0.50%
F3	5.00%	40.00%	9.50%	11.00%	11.00%	17.00%	5.00%	1.00%	0.50%

All ingredients are expressed as percentage by weight (% *w*/*w*). F1, F2, and F3 correspond to different formulations.

**Table 2:** Physicochemical properties of raw propolis and ethanolic propolis extract.

Property	Raw Propolis			Ethanolic Extract of Propolis		
	$\bar{x}$	±	SD	$\bar{x}$	±	SD
Phenolic compounds (mg GAE/g)	6.48 ^a^	±	0.15	17.66 ^b^	±	0.05
Flavonoids (mg QE/g)	6.07 ^a^	±	0.18	13.72 ^b^	±	0.63
Antioxidant capacity by DPPH (µmol TE/g)	45.48 ^a^	±	0.08	102.88 ^b^	±	0.46
L*	33.73 ^a^	±	0.01	41.98 ^b^	±	0.25
a*	3.97 ^a^	±	0.04	16.92 ^b^	±	0.05
b*	9.21 ^a^	±	0.07	66.27 ^b^	±	0.37

Values are expressed as mean ($\bar{x}$) ± standard deviation (SD), *n* = 3. Different letters in the same row indicate statistically significant differences (*p* ≤ 0.05) according to Tukey’s test.

**Table 3:** Factorial design treatments and results of the dependent variables.

Run	A	B	Phenolic Compounds	Flavonoids	DPPH	ABTS	Solubility	Hygroscopicity	Moisture	Bulk Density	Particle Size	ζ Potential
	%	°C	mg GAE/g	mg QE/g	µmol TE/g	µmol TE/g	%	%	%	g/mL	µm	mV
			$\bar{x}$ ± SD	$\bar{x}$ ± SD	$\bar{x}$ ± SD	$\bar{x}$ ± SD	$\bar{x}$ ± SD	$\bar{x}$ ± SD	$\bar{x}$ ± SD	$\bar{x}$ ± SD	$\bar{x}$ ± SD	$\bar{x}$ ± SD
T1	3	120	15.36 ± 0.57	7.77 ± 0.93	56.25 ± 1.98	46.33 ± 0.12	49.64 ± 0.00	15.83 ± 0.68	9.57 ± 0.00	206.71 ± 0.00	6.41 ± 0.03	−34.23 ± 1.55
T2	4	120	10.41 ± 0.06	3.83 ± 0.07	40.79 ± 1.96	25.65 ± 0.71	79.72 ± 0.00	13.08 ± 0.79	5.10 ± 0.10	284.25 ± 0.00	5.39 ± 0.09	−30.30 ± 2.32
T3	5	120	9.84 ± 0.18	3.62 ± 0.21	36.78 ± 1.47	23.09 ± 0.19	66.80 ± 0.00	12.58 ± 1.11	2.70 ± 0.20	274.50 ± 0.00	6.66 ± 0.06	−31.27 ± 0.94
T4	3	130	13.12 ± 0.23	5.18 ± 0.20	60.10 ± 1.99	50.10 ± 0.07	41.49 ± 0.00	15.23 ± 0.07	8.52 ± 0.00	197.07 ± 0.00	6.90 ± 0.09	−37.29 ± 1.89
T5	4	130	11.71 ± 0.12	3.79 ± 0.61	47.34 ± 1.35	46.75 ± 0.07	44.30 ± 0.00	14.65 ± 1.15	7.67 ± 0.00	238.07 ± 0.00	7.80 ± 0.03	−29.25 ± 2.42
T6	5	130	10.77 ± 0.38	2.67 ± 0.07	44.53 ± 1.36	47.98 ± 0.58	47.39 ± 0.00	18.54 ± 0.70	8.62 ± 0.00	239.56 ± 0.00	7.23 ± 0.07	−28.05 ± 0.95
T7	3	140	11.92 ± 0.47	4.31 ± 0.14	59.38 ± 0.35	45.45 ± 0.32	53.90 ± 0.00	20.50 ± 1.67	8.99 ± 0.00	177.75 ± 0.00	6.83 ± 0.04	−34.78 ± 1.57
T8	4	140	10.83 ± 0.28	4.72 ± 0.18	47.04 ± 2.43	25.79 ± 0.21	67.53 ± 0.00	12.72 ± 0.92	5.34 ± 0.46	237.75 ± 0.00	5.64 ± 0.02	−13.20 ± 0.75
T9	5	140	9.16 ± 0.95	2.80 ± 0.48	37.89 ± 2.83	22.19 ± 0.09	56.67 ± 0.00	13.65 ± 0.62	4.29 ± 0.45	234.55 ± 0.00	6.59 ± 0.07	−34.63 ± 0.96

Values are expressed as mean ($\bar{x}$) ± standard deviation (SD), *n* = 3. A represents the percentage of coacervate and B the inlet temperature.

**Table 4:** ANOVA of the factorial design for each dependent variable.

Parameter	*p*-Value Model	R^2^	Intercept	A	B	AB	A^2^	B^2^
Phenolic compounds	<0.0001	0.85	11.39	−1.77 *	−0.62 *	0.69 *	0.71 *	−0.61
*p*-values				<0.0001	0.004	0.01	0.04	0.08
Flavonoids	<0.0001	0.79	4.30	−1.36 *	−0.56 *	0.66 *	0.28	0.63
*p*-values				<0.0001	0.008	0.01	0.39	0.06
DPPH	<0.0001	0.95	47.93	−9.42 *	1.75 *	−0.50	4.10 *	−4.30 *
*p*-values				<0.0001	0.004	0.45	0.0002	0.0001
ABTS	<0.0001	0.86	43.97	−8.10 *	−0.27	−0.003	6.46 *	−16.86 *
*p*-values				<0.0001	0.82	0.99	0.004	<0.0001
Solubility	<0.0001	0.87	51.86	4.31 *	−3.01 *	−3.60 *	−11.20 *	17.98 *
*p*-values				0.001	0.02	0.02	<0.0001	<0.0001
Hygroscopicity	0.008	0.51	14.43	−1.13 *	0.90	−0.90	2.57 *	−1.41
*p*-values				0.04	0.10	0.16	0.008	0.12
Moisture	<0.0001	0.75	7.55	−1.91 *	0.21	0.54	1.08	−2.27 *
*p*-values				<0.0001	0.51	0.17	0.06	0.0003
Bulk density	<0.0001	0.97	246.01	27.85 *	−19.24 *	−2.75	−31.67 *	11.02 *
*p*-values				<0.0001	<0.0001	0.18	<0.0001	0.0007
Particle size	0.0003	0.65	6.98	0.06	0.10	−0.12	0.50 *	−1.06 *
*p*-values				0.62	0.38	0.39	0.02	<0.0001
ζ-Potential	0.0032	0.55	−25.45	2.06	2.20	−0.70	−9.12 *	1.80
*p*-values				0.12	0.09	0.64	0.0003	0.41

A and A^2^ represent the linear and quadratic terms of the coacervate percentage; B and B^2^ the linear and quadratic terms of the inlet temperature; and AB the interaction between both variables. R^2^ indicates the coefficient of determination. An asterisk (*) indicates that the corresponding coefficient in the regression model is statistically significant at *p* ≤ 0.05.

**Table 5:** Optimal parameters of the response variables.

Variable	Experimental Range		Optimal Value			Desirability	
Independent variables	Low	High					
A: Percentage of coacervate (%)	3	5	3.13			0.59	
B: Inlet temperature (°C)	120	140	120				
Dependent variables	Low	High	95% PI low	Experimental value	95% PI high	Predicted value	Assertiveness
Phenolic compounds (mg GAE/g)	9.16	15.36	13.00	14.26 ± 0.15	15.00	14.00	98%
Flavonoids (mg QE/g)	2.67	7.77	5.38	6.09 ± 0.56	7.85	6.61	92%
DPPH (µmol TE/g)	36.78	60.10	49.21	54.10 ± 0.23	56.15	52.68	97%
ABTS (µmol TE/g)	22.19	50.10	31.72	38.15 ± 0.68	46.79	39.26	97%
Solubility (%)	41.49	79.72	50.15	54.67 ± 0.13	65.02	57.59	95%
Hygroscopicity (%)	12.58	20.50	10.93	13.12 ± 0.81	17.56	14.25	92%
Moisture (%)	2.70	9.57	6.00	8.12 ± 0.25	10.02	8.01	99%
Bulk density (g/cm^3^)	177.75	284.25	215.53	229.28 ± 0.86	236.41	225.97	99%
Particle size (µm)	5.39	7.80	5.31	5.68 ± 0.13	6.77	6.04	94%
ζ-Potential (mV)	−37.29	−13.20	−43.10	−33.51 ± 0.77	−27.09	−35.10	95%

PI: Prediction interval (95% confidence). Assertiveness refers to the percentage of experimental values that fall within the prediction interval. Experimental values are expressed as mean ± standard deviation (*n* = 3).

**Table 6:** Phenolic compounds identified in the microcapsules.

Phenolic Compound	Class	Molecular Formula	Molecular Weight (g/mol)	*m*/*z*	Retention Time (min)	Quantity (mg/g)
Gallic acid	Phenolic acid	C_7_H_6_O_5_	170.12	169.10	0.76	0.743 ± 0.01
Catechin	Flavanol	C_15_H_14_O_6_	290.27	291.09	2.18	2.303 ± 0.04
Epicatechin	Flavanol	C_15_H_14_O_6_	290.27	289.02	3.40	2.275 ± 0.03
Epigallocatechin gallate	Flavanol	C_22_H_18_O_11_	458.37	457.08	3.65	0.043 ± 0.01
Rutin	Flavonol (glycoside)	C_27_H_30_O_16_	610.52	609.15	4.97	0.028 ± 0.01
Myricetin	Flavonol	C_15_H_10_O_7_	318.24	317.04	6.76	0.007 ± 0.00
Resveratrol	Stilbene	C_14_H_12_O_3_	228.24	229.08	7.39	0.181 ± 0.02
Quercetin	Flavonol	C_15_H_10_O_7_	302.24	301.04	8.36	0.005 ± 0.00
Kaempferol	Flavonol	C_15_H_10_O_6_	286.23	285.05	9.12	0.181 ± 0.02

Phenolic compounds were identified based on retention times (RTs), UV absorption spectra, molecular weights, and mass-to-charge (*m*/*z*) ratios, using certified external standards. Quantification was performed by UPLC-PDA-QDa in triplicate, based on compound-specific calibration curves, and the results are expressed as mean ± standard deviation in mg/g of dry microcapsules.

**Table 7:** Texture profile analysis in the gummy candies.

Formulation	Hardness (N)			Cohesiveness			Adhesiveness (g)			Springiness (mm)			Resilience		
	$\bar{x}$	±	SD	$\bar{x}$	±	SD	$\bar{x}$	±	SD	$\bar{x}$	±	SD	$\bar{x}$	±	SD
F1	1.49 ^ab^	±	0.17	0.92 ^ab^	±	0.03	3.40 ^a^	±	0.80	3.05 ^a^	±	0.03	0.51 ^a^	±	0.04
F2	1.41 ^a^	±	0.33	0.97 ^a^	±	0.08	4.07 ^a^	±	0.33	3.12 ^a^	±	0.03	0.56 ^a^	±	0.01
F3	2.58 ^b^	±	0.89	0.87 ^b^	±	0.02	1.53 ^b^	±	0.31	3.02 ^a^	±	0.12	0.50 ^a^	±	0.03

Values are expressed as arithmetic mean ($\bar{x}$) ± standard deviation (SD), *n* = 3. Different letters in the same column indicate statistically significant differences (*p* ≤ 0.05) according to Tukey’s test.

## Data Availability

They are available in the same article.

## References

[1] Braakhuis A. (2019). Evidence on the Health Benefits of Supplemental Propolis. Nutrients.

[2] Chavda V.P., Vuppu S., Balar P.C., Mishra T., Bezbaruah R., Teli D., Sharma N., Alom S. (2024). Propolis in the management of cardiovascular disease. Int. J. Biol. Macromol..

[3] Pasupuleti V.R., Sammugam L., Ramesh N., Gan S.H. (2017). Honey, Propolis, and Royal Jelly: A Comprehensive Review of Their Biological Actions and Health Benefits. Oxidative Med. Cell. Longev..

[4] Ligarda-Samanez C.A., Choque-Quispe D., Moscoso-Moscoso E., Huamán-Carrión M.L., Ramos-Pacheco B.S., Peralta-Guevara D.E., Cruz G.D., Martínez-Huamán E.L., Arévalo-Quijano J.C., Muñoz-Saenz J.C. (2022). Obtaining and Characterizing Andean Multi-Floral Propolis Nanoencapsulates in Polymeric Matrices. Foods.

[5] Jansen-Alves C., Martins Fonseca L., Doring Krumreich F., Zavareze E.D.R. (2023). Applications of propolis encapsulation in food products. J. Microencapsul..

[6] Ligarda-Samanez C.A., Choque-Quispe D., Moscoso-Moscoso E., Huamán-Carrión M.L., Ramos-Pacheco B.S., De la Cruz G., Arévalo-Quijano J.C., Muñoz-Saenz J.C., Muñoz-Melgarejo M., Quispe-Quezada U.R. (2023). Microencapsulation of Propolis and Honey Using Mixtures of Maltodextrin/Tara Gum and Modified Native Potato Starch/Tara Gum. Foods.

[7] Tavares L., Smaoui S., Lima P.S., de Oliveira M.M., Santos L. (2022). Propolis: Encapsulation and application in the food and pharmaceutical industries. Trends Food Sci. Technol..

[8] Maroof K., Lee R.S.F., Siow S.F., Gan S.H. (2022). Microencapsulation of propolis by spray drying: A review. Dry. Technol..

[9] Muhoza B., Yuyang H., Uriho A., Harindintwali J.D., Liu Q., Li Y. (2023). Spray-and freeze-drying of microcapsules prepared by complex coacervation method: A review. Food Hydrocoll..

[10] Sukri N., Marin P.T.T., Mahani, Nurhadi B. (2023). Characteristics of propolis encapsulated with gelatin and sodium alginate by complex coacervation method. Int. J. Food Prop..

[11] Hernández-Nava R., López-Malo A., Palou E., Ramírez-Corona N., Jiménez-Munguía M.T. (2020). Encapsulation of oregano essential oil (Origanum vulgare) by complex coacervation between gelatin and chia mucilage and its properties after spray drying. Food Hydrocoll..

[12] Hernández-Nava R., López-Malo A., Palou E., Ramírez-Corona N., Jiménez-Munguía M.T. (2019). Complex Coacervation Between Gelatin and Chia Mucilage as an Alternative of Encapsulating Agents. J. Food Sci..

[13] Bustamante M., Laurie-Martínez L., Vergara D., Campos-Vega R., Rubilar M., Shene C. (2020). Effect of Three Polysaccharides (Inulin, and Mucilage from Chia and Flax Seeds) on the Survival of Probiotic Bacteria Encapsulated by Spray Drying. Appl. Sci..

[14] Busch V.M., Pereyra-Gonzalez A., Šegatin N., Santagapita P.R., Poklar Ulrih N., Buera M.P. (2017). Propolis encapsulation by spray drying: Characterization and stability. LWT.

[15] Kurek-Górecka A., Kara Y., Pokajewicz K., Ben Hammouda I., Wieczorek P.P., Marciniak D., Balwierz R., Kłósek M., Czuba Z.P., Kolaylı S. (2025). Phenolic content, volatile compounds and antioxidant activity in pooled propolis samples from Turkey and Poland. Eur. Food Res. Technol..

[16] Ligarda-Samanez C.A., Choque-Quispe D., Moscoso-Moscoso E., Pozo L.M.F., Ramos-Pacheco B.S., Palomino-Rincón H., Gutiérrez R.J.G., Peralta-Guevara D.E. (2023). Effect of Inlet Air Temperature and Quinoa Starch/Gum Arabic Ratio on Nanoencapsulation of Bioactive Compounds from Andean Potato Cultivars by Spray-Drying. Molecules.

[17] Ligarda-Samanez C.A., Choque-Quispe D., Moscoso-Moscoso E., Palomino-Rincón H., Taipe-Pardo F., Aguirre Landa J.P., Arévalo-Quijano J.C., Muñoz-Saenz J.C., Quispe-Quezada U.R., Huamán-Carrión M.L. (2023). Nanoencapsulation of Phenolic Extracts from Native Potato Clones (*Solanum tuberosum* spp. *andigena*) by Spray Drying. Molecules.

[18] Ligarda-Samanez C.A., Palomino-Rincón H., Choque-Quispe D., Moscoso-Moscoso E., Arévalo-Quijano J.C., Huamán-Carrión M.L., Quispe-Quezada U.R., Muñoz-Saenz J.C., Gutiérrez-Gómez E., Cabel-Moscoso D.J. (2023). Bioactive Compounds and Sensory Quality in Chips of Native Potato Clones (*Solanum tuberosum* spp. *andigena*) Grown in the High Andean Region of PERU. Foods.

[19] Moscoso-Moscoso E., Ligarda-Samanez C.A., Choque-Quispe D., Huamán-Carrión M.L., Arévalo-Quijano J.C., De la Cruz G., Luciano-Alipio R., Calsina Ponce W.C., Sucari-León R., Quispe-Quezada U.R. (2024). Preliminary Assessment of Tara Gum as a Wall Material: Physicochemical, Structural, Thermal, and Rheological Analyses of Different Drying Methods. Polymers.

[20] Berretta A.A., Zamarrenho L.G., Correa J.A., De Lima J.A., Borini G.B., Ambrósio S.R., Barud H.D., Bastos J.K., De Jong D. (2023). Development and Characterization of New Green Propolis Extract Formulations as Promising Candidates to Substitute for Green Propolis Hydroalcoholic Extract. Molecules.

[21] Andrade J.K.S., Denadai M., Andrade G.R.S., da Cunha Nascimento C., Barbosa P.F., Jesus M.S., Narain N. (2018). Development and characterization of microencapsules containing spray dried powder obtained from Brazilian brown, green and red propolis. Food Res. Int..

[22] Andrade J.K.S., Denadai M., de Oliveira C.S., Nunes M.L., Narain N. (2017). Evaluation of bioactive compounds potential and antioxidant activity of brown, green and red propolis from Brazilian northeast region. Food Res. Int..

[23] Ligarda-Samanez C.A., Villano-Limache E., Pichihua-Oscco W., Choque-Quispe D., Sucari-León R., Calderón Huamaní D.F., Cruz G.D., Luciano-Alipio R., Calsina Ponce W.C., Aroquipa-Durán Y. (2025). Physicochemical and Sensory Evaluation of Gummy Candies Fortified with Microcapsules of Guinea Pig (*Cavia porcellus*) Blood Erythrocytes and Tumbo (*Passiflora tarminiana*) Juice. Appl. Sci..

[24] Tumbarski Y., Ivanov I., Todorova M., Apostolova S., Tzoneva R., Nikolova K. (2025). Phenolic Content, Antioxidant Activity and In Vitro Anti-Inflammatory and Antitumor Potential of Selected Bulgarian Propolis Samples. Biomedicines.

[25] Rosales-Chimal S., Navarro-Cortez R.O., Bello-Perez L.A., Vargas-Torres A., Palma-Rodríguez H.M. (2023). Optimal conditions for anthocyanin extract microencapsulation in taro starch: Physicochemical characterization and bioaccessibility in gastrointestinal conditions. Int. J. Biol. Macromol..

[26] Das A.B., Goud V.V., Das C. (2019). Microencapsulation of anthocyanin extract from purple rice bran using modified rice starch and its effect on rice dough rheology. Int. J. Biol. Macromol..

[27] Pedrozo R.C., Antônio E., Khalil N.M., Mainardes R.M. (2020). Bovine serum albumin-based nanoparticles containing the flavonoid rutin produced by nano spray drying. Braz. J. Pharm. Sci..

[28] Mousavi Kalajahi S.E., Ghandiha S. (2022). Optimization of spray drying parameters for encapsulation of Nettle (*Urtica dioica* L.) extract. LWT.

[29] Kyriakoudi A., Tsimidou M.Z. (2018). Properties of encapsulated saffron extracts in maltodextrin using the Büchi B-90 nano spray-dryer. Food Chem..

[30] Nayak C.A., Rastogi N.K. (2010). Effect of Selected Additives on Microencapsulation of Anthocyanin by Spray Drying. Dry. Technol..

[31] Vergara C., Pino M.T., Zamora O., Parada J., Pérez R., Uribe M., Kalazich J. (2020). Microencapsulation of Anthocyanin Extracted from Purple Flesh Cultivated Potatoes by Spray Drying and Its Effects on In Vitro Gastrointestinal Digestion. Molecules.

[32] Bezerra M.A., Santelli R.E., Oliveira E.P., Villar L.S., Escaleira L.A. (2008). Response surface methodology (RSM) as a tool for optimization in analytical chemistry. Talanta.

[33] Duca A., Sturza A., Moacă E.-A., Negrea M., Lalescu V.-D., Lungeanu D., Dehelean C.-A., Muntean D.-M., Alexa E. (2019). Identification of Resveratrol as Bioactive Compound of Propolis from Western Romania and Characterization of Phenolic Profile and Antioxidant Activity of Ethanolic Extracts. Molecules.

[34] Volpi N. (2004). Separation of flavonoids and phenolic acids from propolis by capillary zone electrophoresis. Electrophoresis.

[35] Rocha V.M., Portela R.W., Lacerda L.E., Sokolonski A.R., de Souza C.O., dos Anjos J.P., Nascimento R.Q., Umsza-Guez M.A. (2023). Propolis from different Brazilian stingless bee species: Phenolic composition and antimicrobial activity. Food Prod. Process. Nutr..

[36] Huang S., Zhang C.-P., Wang K., Li G.Q., Hu F.-L. (2014). Recent Advances in the Chemical Composition of Propolis. Molecules.

[37] Freitas M.O., Ponte F.A., Lima M.A.S., Silveira E.R. (2008). Flavonoids and triterpenes from the nest of the stingless bee Trigona spinipes. J. Braz. Chem. Soc..

[38] Heng D., Lee S.H., Ng W.K., Tan R.B.H. (2011). The nano spray dryer B-90. Expert Opin. Drug Deliv..

[39] Delia S.-C., Chávez G.M., León-Martínez Frank M., Araceli S.-G.P., Irais A.-L., Franco A.-A. (2019). Spray drying microencapsulation of betalain rich extracts from Escontria chiotilla and Stenocereus queretaroensis fruits using cactus mucilage. Food Chem..

[40] Pashazadeh H., Zannou O., Ghellam M., Koca I., Galanakis C.M., Aldawoud T.M.S. (2021). Optimization and Encapsulation of Phenolic Compounds Extracted from Maize Waste by Freeze-Drying, Spray-Drying, and Microwave-Drying Using Maltodextrin. Foods.

[41] Fredes C., Becerra C., Parada J., Robert P. (2018). The Microencapsulation of Maqui (*Aristotelia chilensis* (Mol.) Stuntz) Juice by Spray-Drying and Freeze-Drying Produces Powders with Similar Anthocyanin Stability and Bioaccessibility. Molecules.

[42] Cedeño-Pinos C., Marcucci M.C., Bañón S. (2021). Contribution of Green Propolis to the Antioxidant, Physical, and Sensory Properties of Fruity Jelly Candies Made with Sugars or Fructans. Foods.

[43] Calderón-Oliver M., Ponce-Alquicira E. (2022). The Role of Microencapsulation in Food Application. Molecules.

[44] Otálora M.C., Wilches-Torres A., Gómez Castaño J.A. (2023). Microencapsulation of Betaxanthin Pigments from Pitahaya (*Hylocereus megalanthus*) By-Products: Characterization, Food Application, Stability, and In Vitro Gastrointestinal Digestion. Foods.

[45] González-Montiel L., Miranda-Altamirano D., Bautista-Marcial A.S., Güemes-Vera N., Soto-Simental S., Franco-Fernández M.J., Sánchez-Hernández C., Campos-Pastelín J.M. (2019). Análisis de perfil de textura y color en gomitas elaboradas a partir de una deccoción de plantas medicinales. Investig. Desarro. Cienc. Tecnol. Aliment..

